# A Virtual, Randomized, Control Trial of a Digital Therapeutic for Speech, Language, and Cognitive Intervention in Post-stroke Persons With Aphasia

**DOI:** 10.3389/fneur.2021.626780

**Published:** 2021-02-12

**Authors:** Michelle Braley, Jordyn Sims Pierce, Sadhvi Saxena, Emily De Oliveira, Laura Taraboanta, Veera Anantha, Shaheen E. Lakhan, Swathi Kiran

**Affiliations:** ^1^Department of Communication Sciences and Disorders, MGH Institute of Health Professions, Charlestown, MA, United States; ^2^Spaulding Rehabilitation Hospital, Charlestown, MA, United States; ^3^The Learning Corp, Newton, MA, United States; ^4^Constant Therapy Health, Lexington, MA, United States; ^5^Pierce Health Consultants, Lenox, MA, United States; ^6^Department of Neurology, Johns Hopkins School of Medicine, Baltimore, MD, United States; ^7^Definitive Healthcare, Boston, MA, United States; ^8^Global Neuroscience Initiative Foundation, Boston, MA, United States; ^9^Virginia Tech, Blacksburg, VA, United States; ^10^Sargent College of Health and Rehabilitation Sciences, Boston University, Boston, MA, United States

**Keywords:** tele-neurorehabilitation, post-stroke aphasia, virtual treatment, language outcomes, remote assessment

## Abstract

**Background:** Post-stroke aphasia is a chronic condition that impacts people's daily functioning and communication for many years after a stroke. Even though these individuals require sustained rehabilitation, they face extra burdens to access care due to shortages in qualified clinicians, insurance limitations and geographic access. There is a need to research alternative means to access intervention remotely, such as in the case of this study using a digital therapeutic.

**Objective:** To assess the feasibility and clinical efficacy of a virtual speech, language, and cognitive digital therapeutic for individuals with post-stroke aphasia relative to standard of care.

**Methods:** Thirty two participants completed the study (experimental: average age 59.8 years, 7 female, 10 male, average education: 15.8 years, time post-stroke: 53 months, 15 right handed, 2 left handed; control: average age 64.2 years, 7 female, 8 male, average education: 15.3 years, time post-stroke: 36.1 months, 14 right handed, 1 left handed). Patients in the experimental group received 10 weeks of treatment using a digital therapeutic, Constant Therapy-Research (CT-R), for speech, language, and cognitive therapy, which provides evidence-based, targeted therapy with immediate feedback for users that adjusts therapy difficulty based on their performance. Patients in the control group completed standard of care (SOC) speech-language pathology workbook pages.

**Results:** This study provides Class II evidence that with the starting baseline WAB-AQ score, adjusted by −0.69 for every year of age, and by 0.122 for every month since stroke, participants in the CT-R group had WAB-AQ scores 6.43 higher than the workbook group at the end of treatment. Additionally, secondary outcome measures included the WAB-Language Quotient, WAB-Cognitive Quotient, Brief Test of Adult Cognition by Telephone (BTACT), and Stroke and Aphasia Quality of Life Scale 39 (SAQOL-39), with significant changes in BTACT verbal fluency subtest and the SAQOL-39 communication and energy scores for both groups.

**Conclusions:** Overall, this study demonstrates the feasibility of a fully virtual trial for patients with post-stroke aphasia, especially given the ongoing COVID19 pandemic, as well as a safe, tolerable, and efficacious digital therapeutic for language/cognitive rehabilitation.

**Clinical Trial Registration:** www.ClinicalTrials.gov, identifier NCT04488029.

## Introduction

According to the Centers for Disease Control and Prevention (CDC), every year an estimated 795,000 Americans will have a stroke, and more than 180,000 will be left with communication disorders such as aphasia (1, 2). Aphasia can impact a person's ability to understand and follow instructions or read a prescription label. It can isolate a person from their family and friends, impacting their sense of self and bringing with it a myriad of other loneliness-related health risks (3).

Aphasia is a chronic condition that requires ongoing rehabilitation (4). It was once thought that recovery only occurred in the first year of a stroke; however, a growing body of evidence shows that people with aphasia (PWA) can continue to improve with ongoing rehabilitation even many years after their injury (4, 5). A recent Cochrane review suggests that functional communication significantly improves when one receives speech-language therapy at a high intensity, across several sessions, or over a long period of time (6). Despite the evidence that supports the need for ongoing therapy, there are not enough therapists who can treat post-stroke aphasia. The expectation for therapists to provide therapy five times per week during the chronic phase of care is simply not feasible. In addition to limited access to therapists, other barriers that patients experience include limited insurance coverage, lack of transportation, distant geography, schedule constraints, and fatigue (7). As a result, rehabilitation for aphasia patients is quite fragmented (8), or insufficient, especially for stroke survivors living in the community but not in active therapy (4) which ultimately leads to worse patient outcomes, especially when they can benefit from ongoing therapy post-discharge. Since the COVID19 pandemic began, individuals with aphasia have faced even greater hurdles in accessing the care they need due to safety restrictions exacerbating disparities in healthcare for these individuals (9).

Teletherapy, or technology assisted/delivered therapy, provides an alternative to the brick-and-mortar approach of delivering rehabilitation services (10–14). In such an approach, therapy is delivered via a computer and over the internet asynchronously but follows the same basic principles of traditional person-to-person rehabilitation. A clinician can also supervise teletherapy sessions remotely. Early indications illustrate that such technology would afford PWA greater opportunity for consistent and intensive practice, especially when coming into the clinic is not feasible (15, 16). Further, teletherapy may also allow long-term continued rehabilitation to be more accessible for PWA. While some aphasia research highlights the limitations in using technology with this population (17, 18), other research demonstrates positive outcomes in improving language skills with technology (19–22). Recent systematic reviews have examined different technology-based rehabilitation delivery options for both cognitive deficits (23, 24) and language deficits (25–28). Further, a recent RCT specifically compared treatment outcomes for PWA receiving self-managed computerized speech therapy relative to other control treatments (20). In this study, 278 PWA were assigned to either daily self-managed computerized speech language therapy plus usual care (experimental, CSLT group), usual care (usual care group), or attention control plus usual care (attention control group). Treatment was completed for 6 months and results showed that the experimental group receiving computerized therapy (CSLT) demonstrated significantly higher gains in trained word finding relative to the two control groups, however, there was no evidence of generalization to untrained words. Further, there were no differences in functional communication or participants' perception of their own communication or participation across the three intervention groups. Nonetheless, these results add to the emerging premise that remote or home-based computerized therapy can be a valid approach to deliver rehabilitation to individuals with post-stroke aphasia.

In our prior work with teletherapy, we have examined the feasibility and clinical efficacy of Constant Therapy-Research (CT-R^1^), a digital therapeutic software program accessible through a tablet (29–31). CT-R is a prototype based on the commercially available Constant Therapy product. In a previous study, 51 subjects (42 experimental, 9 control) utilized the Constant Therapy software platform under the systematic monitoring and guidance from their clinician during weekly in-clinic sessions (29). The experimental group had access to Constant Therapy both at home and during in-clinic sessions, while the control group only utilized the application during in-clinic sessions. After 10 weeks of intervention, experimental participants were significantly more engaged in their therapy and practiced an additional 4 h per week on average compared to the in-clinic therapy sessions, where participants received an average of 40 min per week. In addition, experimental participants showed significantly more improvements on Constant Therapy tasks and on standardized language and cognitive tests than control participants. Separately, in a retrospective analysis of Constant Therapy home users vs. clinic users (31), both home and clinic users required roughly the same amount of practice to successfully complete cognitive and language tasks, but users who had on-demand access to therapy on their tablet mastered tasks in a median of 6 days, while those with only in-clinic access mastered tasks in a median of 12 days. Further, users who had access to digital therapy at home practiced at least every 2 days, while clinic users practiced in the clinic just once every 5 days. These findings suggest that Constant Therapy users were able to practice structured therapy at home, which provided them with greater practice and greater intensity of therapy than patients receiving the same therapy in clinic. These studies also highlighted the potential for a home-based therapy program for patients who are unable to receive consistent in-clinic therapy.

The primary objective of this study was to examine the efficacy of CT-R practiced under the remote-guidance of a study personnel when compared to an active control group that practiced aphasia therapy workbooks. Our rationale was that self-management of home-based therapy under remote-guidance that included an individualized therapy protocol would lead to increased adherence and compliance of home practice, and ultimately improved language and cognitive skills. We conducted a Phase II, randomized decentralized (virtual) trial, in which 36 participants (stroke survivors with aphasia) received either language therapy at home delivered through CT-R or practiced aphasia therapy workbooks at home. Both groups received baseline and follow-up assessments, as well as periodic therapy check-in sessions, through video conference sessions. The primary outcome of the study was change in the Western Aphasia Battery-R Aphasia Quotient (WAB-R AQ) (32). The primary hypothesis was that self-managed, digital therapy under remote supervision would result in systematic and structured reinforcement-based practice of impairment based therapy, which would ultimately lead to greater language outcomes, as compared to the control group that did not receive this systematic structured practice. Additionally, to the best of our knowledge, this is the first fully virtual language therapy study for individuals with aphasia.

## Methods

### Recruitment

As this was a completely virtual study, participants were recruited from the United States and Canada from March 2019 to November 2019. The following were sources of participant recruitment: (a) consumers who had downloaded the commercially available Constant Therapy app but not signed up for an account, (b) social media groups focused on recovery from aphasia, and (c) referrals from SLPs who had discharged clients from their service. Recruitment was conducted via email, video advertising, flyers, and social media posts.

### Participants

Inclusion criteria included (a) diagnosis of stroke involving a hemorrhage or ischemic event, resulting in speech, language, and/or cognitive deficits as confirmed by medical records; (b) time post-stroke of at least 4 months prior to enrollment; (c) having been discharged from the hospital or rehabilitation center; (d) being aged 18 years or older at the time of consent; (e) being a fluent English speaker prior to stroke; (f) having confirmed aphasia based on the Western Aphasia Battery, Revised (WAB-R) (32) Aphasia Quotient with a score of 90 or lower (normal cutoff score is 93.8), and (g) the presence of a family member or caregiver willing and able to provide assistance during the duration of the study period.

Exclusionary criteria included (a) comorbid neurological conditions that could impair study performance in the opinion of research staff (either a certified Speech-Language Pathologist or a trained Research Assistant), (b) requiring inpatient care or acute care at the time of the study, (c) concurrently undergoing one-on-one individual therapy at a hospital or rehabilitation facility, university, or at home, (d) presence of severe apraxia of speech or severe dysarthria of speech based on clinical screening, (e) comorbid psychiatric conditions that could impair study participation in the opinion of study staff, and (f) uncorrected vision or hearing loss impairing study participation.

**Figure 1:**
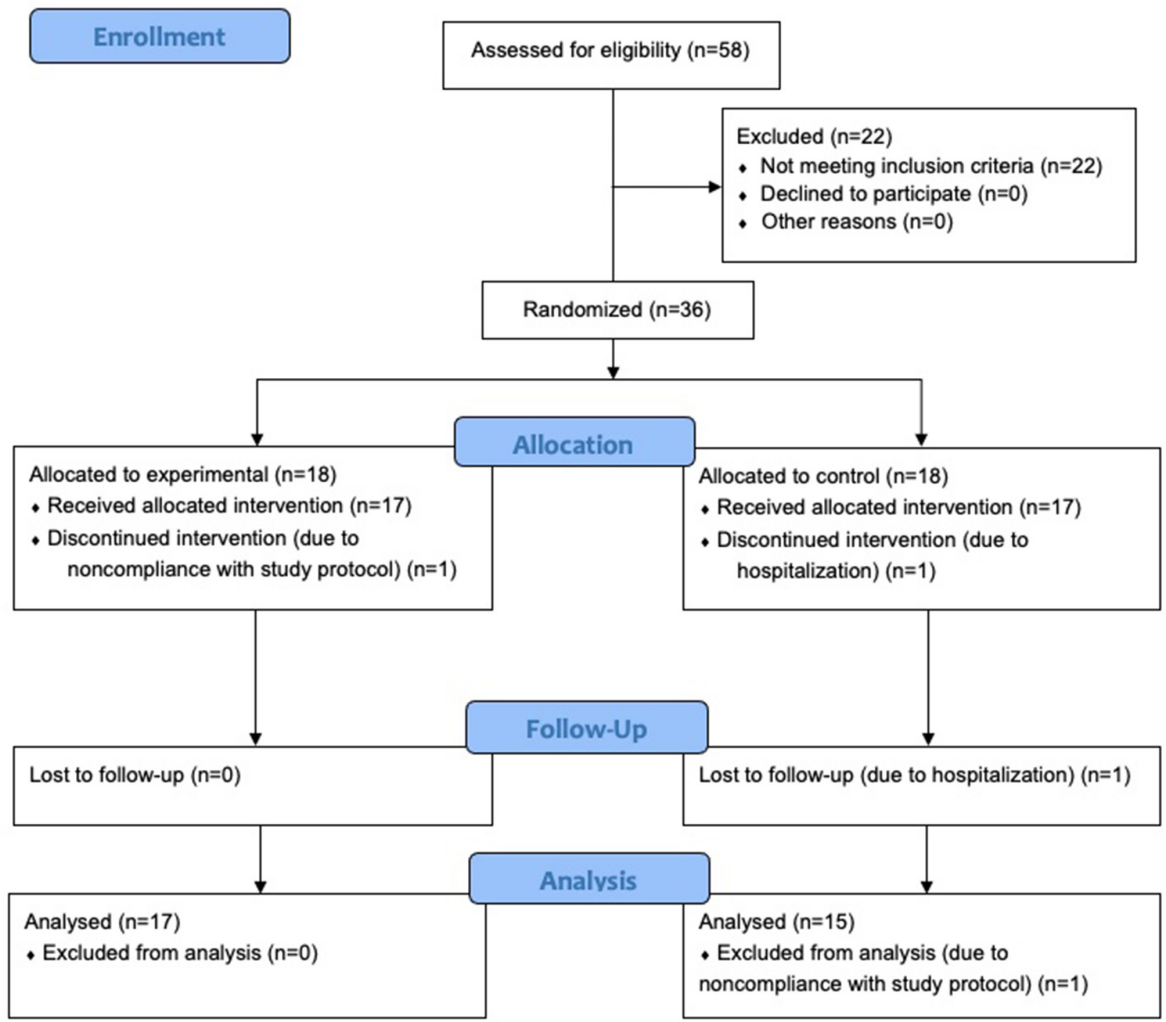
Consort participant flow diagram.

A pre-screening phone call was conducted by the research staff with the participant and caregiver to discuss the details of the study and participant characteristics. Then, each participant was mailed materials that included: an iPad tablet, WAB-R assessment items, informed consent and medical release forms, and a pre-addressed and stamped envelope to return consent and release forms. Following informed consent, participants were evaluated utilizing Part 1 of the WAB-R following procedures for videoconference assessment (33). If all eligibility criteria were met, the participant was enrolled in the study. Of the 58 participants that were screened against eligibility criteria, 36 were enrolled and 32 completed the study (see Figure 1). Of those who completed the study, the mean age of participants was 61 years (*SD* = 10), 18 participants were male, the average time post-stroke was 46 months (*SD* = 47), and the mean years in education was 15 years (*SD* = 2.6). As noted above, all participants completed all parts of the study from their homes.

### Primary and Secondary Outcome Measures

The primary outcome measure utilized was the Western Aphasia Battery, Revised (WAB-R) Aphasia Quotient (WAB-AQ) (32). The WAB-R is a standardized tool that assesses language and cognitive skills and provides scores quantifying the impact of a stroke on those skills. The WAB-AQ from the WAB-R includes segments from Part 1 of the assessment, evaluating fluency and information content within spontaneous speech, auditory comprehension, naming, and repetition. The Language and Cortical Quotients obtained from the WAB-R (WAB-LQ and WAB-CQ) Parts 1 and 2 were utilized as secondary outcome measures. Part 2 of the WAB-R includes reading, writing, apraxia, constructional, visuospatial, and calculation sections.

Additionally, secondary measures included scores on the Brief Test of Adult Cognition by Telephone (BTACT) (34, 35), a brief, remote, cognitive assessment that evaluates memory for and judgments about words and numbers (including recall tasks, both immediate and short term, category fluency, and number reasoning and manipulation tasks), and the Stroke and Aphasia Quality of Life Scale 39 (SAQOL-39) (36, 37). The SAQOL-39 is a structured quality of life questionnaire administered to either a patient or a caregiver to assess the impact of a stroke on daily activities, communication, emotions, and family and social life by asking patients or caregivers to complete a 5-point rating scale in response to specific questions focusing on the past week alone. All the above measures were chosen based on prior evidence for having been administered remotely, either by videoconference or by phone (33, 35, 38, 39).

### Assessments

Following informed consent, and the administration of the WAB-R, if the participant met eligibility criteria of an aphasia quotient of 90 or below, the remainder of the assessments were completed. For participants who were identified with potential dysarthria or apraxia, the Screen for Dysarthria and Apraxia of Speech was then completed to exclude any participant that received a “severe” score on the three features of diadochokinesis, word length, and oral apraxia. Subsequently, assessment continued on with the second portion of the WAB-R, the BTACT (34) and SAQOL-39 (39). When needed, the SAQOL-39 proxy form was provided to the caregiver to complete on behalf of the participant. As the clinician was remote, a caregiver was present with the participant during the virtual assessment to facilitate video conferencing setup and test administration. At the start of the assessment, a brief training was provided to the participant and caregiver on the videoconferencing technology. Instruction was provided to the caregiver to refrain from providing cues or hints to test items. At the conclusion of the assessment, a follow-up phone call was scheduled with the participant and caregiver within the same week to discuss next steps for participation in the study. See Table 1 for demographic information on study participants, and Table 2 for pre-treatment assessment data.

**Table 1:** Participant demographic information.

**Demographic information**	**Experimental group**	**Control group**
*N*	17	15
Age (years)	58.9 (10)	64.2 (9.9)
Sex (female/male)	7/10	7/8
Education (years)	15.8 (2.7)	15.3 (2.5)
Time post-stroke (months)	53.0 (56)	38.1 (32)
Handedness (right/left)	15/2	14/1

*Means and standard deviations (provided in parenthesis)*.

**Table 2:** Pre-treatment and post-treatment assessment scores for WAB, BTACT, and SAQOL39.

**Outcome measures**	**Experimental**		**Control**	
	**Pre**	**Post**	**Pre**	**Post**
***Western Aphasia Battery, Revised (WAB-R)***				
**Aphasia Quotient**	**61.62 (24.28)**	**68.37 (26.24)**	**66.02 (19.08)**	**66.40 (20.22)**
Spontaneous speech	11.41 (5.33)	12.76 (5.78)	12.40 (3.38)	12.07 (4.01)
Auditory verbal comprehension	155.18 (38.02)	167.24 (33.96)	152.33 (38.78)	156.80 (35.99)
Repetition	50.76 (28.79)	60.88 (29.02)	62.60 (26.85)	66.07 (26.36)
Naming and word finding	65.65 (31.07)	69.71 (32.58)	67.33 (23.13)	66.87 (25.14)
**Language Quotient**	**64.64 (25.49)**	**69.15 (25.33)**	**66.01 (21.72)**	**66.57 (21.51)**
Reading	70.47 (28.65)	74.47 (23.82)	69.00 (25.13)	70.20 (25.10)
Writing	59.88 (36.73)	58.53 (33.82)	57.90 (32.05)	57.47 (27.50)
**Cortical Quotient**	**67.69 (22.68)**	**72.38 (23.27)**	**68.81 (18.71)**	**69.59 (19.00)**
Apraxia	49.94 (12.50)	51.71 (11.74)	50.87 (9.50)	50.53 (9.52)
Constructional, visuospatial, and calculation	77.62 (18.68)	79.18 (22.13)	70.20 (17.09)	73.60 (16.66)
***Brief Test of Adult Cognition by Telephone (BTACT)***				
Immediate recall	2.21 (1.81)	3.15 (1.63)	2.07 (1.44)	2.07 (1.71)
Digit span backwards	2.57 (1.16)	2.71 (1.14)	1.73 (1.22)	2.20 (1.37)
Fluency	9.71 (5.51)	11.43 (6.17)	6.87 (5.11)	8.00 (6.93)
Number series	0.64 (1.15)	0.93 (1.44)	0.33 (0.62)	0.33 (0.82)
Backward counting	10.91 (11.32)	10.93(12.28)	7.27 (10.64)	8.13 (9.78)
Delayed recall	0.71 (1.44)	1.21 (1.89)	1.07 (1.28)	1.20 (1.32)
***Stroke and Aphasia Quality of Life Scale−39 (SAQOL-39)***				
Mean	3.53 (0.54)	3.77 (0.56)	3.57 (0.58)	3.66 (0.70)
Physical	4.08 (0.70)	4.16 (0.61)	3.92 (0.98)	3.89 (0.95)
Communication	2.70 (0.64)	2.96 (0.71)	2.59 (0.82)	2.74 (0.86)
Psychosocial	3.54 (0.81)	3.81 (0.90)	3.75 (0.54)	3.64 (1.05)
Energy	2.88 (0.97)	3.48 (1.11)	3.54 (1.09)	3.63 (0.91)

*Means and standard deviations (in parenthesis)*.

### Study Design

Given the preliminary nature of the treatment protocol in this study, one of the purposes of this study was to generate effect sizes for future definitive clinical trials. Hence, this paper does not report apriori sample size estimates. The study participation lasted ~14 weeks, which included: recruitment and baseline assessment (−2 to 0 weeks), treatment period (0–10 weeks), bi-weekly check-ins (weeks 2, 4, 6, and 8) and follow-up assessment (10–12 weeks). As noted, the entire study, including recruitment, enrollment, and study interventions was conducted remotely (i.e., at participant homes). The primary and secondary outcomes (WAB-R, BTACT, and SAQOL-39) were remotely administered at baseline (Week 0) and post-intervention (Week 10–12). After pre-assessment, stratified randomization was applied to assign participants into one of two groups (experimental or control) to balance for overall aphasia severity (WAB-AQ). Thus, the design was a parallel 1:1 allocation ratio with an initial random-numbers table to generate an allocation sequence that was then balanced for aphasia severity during assignments. Given the nature of the two interventions and the bi-weekly check-ins that relied on the nature of intervention, no attempt was made to blind participants or experimenters in the study. However, pre-treatment and post-treatment assessments were administrated by a team of study staff randomly assigned to participants from either group. Further, fidelity and reliability in testing administration was conducted and is described below. To encourage participation and retention, tablets were supplied with active cellular data plans, and training for how to use the tablet and app was provided to the participant and caregiver as needed.

#### Experimental Group (CT-R)

**Figure 2:**
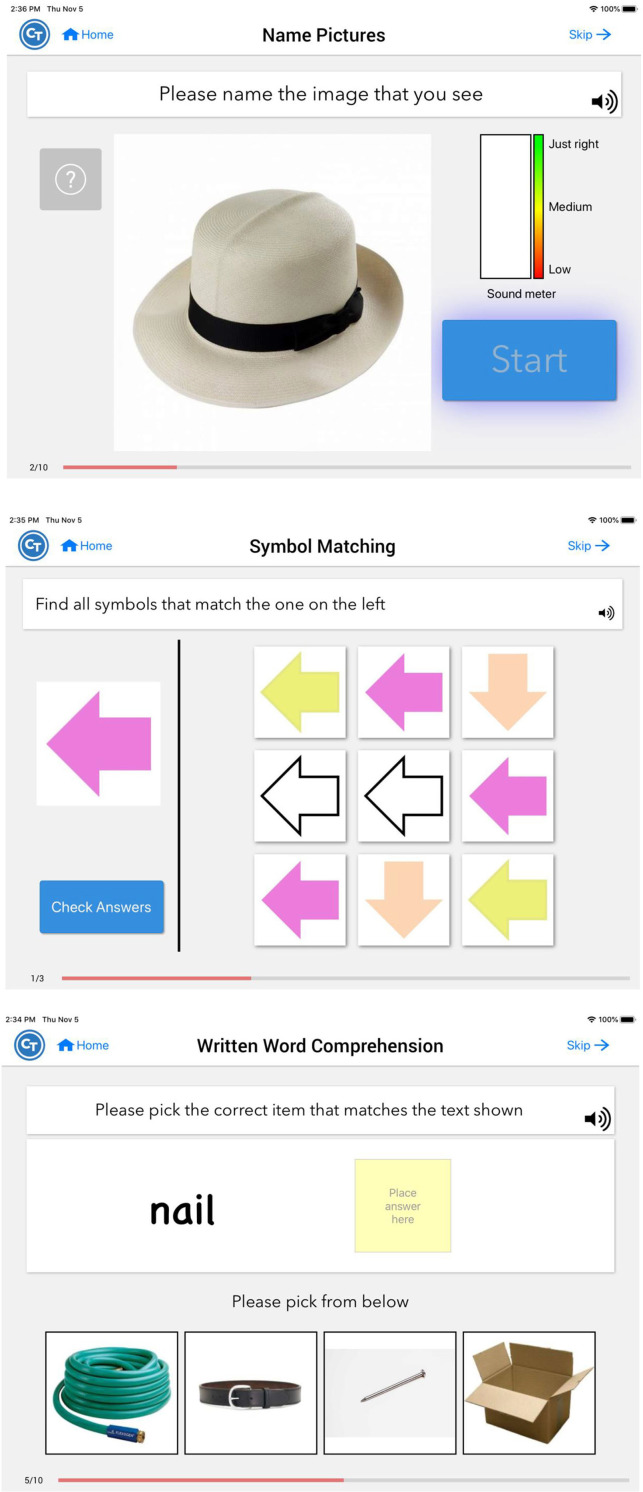
Screenshots of CT-R software.

Participants were instructed to use a provisioned tablet with the app pre-installed. Constant Therapy (www.constanttherapy.com) provides systematic and structured therapy analogous to what is typically provided by a speech-language pathologist (SLP) that can be accessed by the patient from any location using a supported device. The NeuroPerformance Engine (NPE), a patented technology, enables the product to optimize therapeutic delivery (i.e., progress across tasks or reduce the level of difficulty) based on a patient's individual performance. An initial homework schedule was created and assigned by the study team according to each individual's WAB-R performance with guidelines that were standardized based on score cut-offs across participants. From that point, the individual was advanced via NPE algorithm using the library of therapy exercises within the CT-R app. Across exercises, there are over 100,000 stimuli within 350+ levels of difficulty spanning 9 different cognitive, speech and language domains (see Figure 2). Participants were instructed to use CT-R for at least 30 min a day and at least 5 days a week. CT-R tracked usage of the program so that research staff could access automated reporting of participant use to monitor participant adherence to the treatment program (29, 31).

#### Control Group (Workbooks)

Participants were provided with a regime of standard, paper workbooks (40–44) used for homework practice, a substantial modification from the workbooks used in the usual care control group in the BIG CACTUS study (20) that used crossword puzzles. The progression of homework went from Workbook for Aphasia (40) to the Speech Therapy Aphasia Rehabilitation Workbooks (41–43) or the Workbook of Activities for Language and Cognition (WALC 1) (44) based on feedback about difficulty. Control participants were instructed to complete at least 1 exercise within the workbook at least 5 days a week.

On a bi-weekly basis from Weeks 2 through 8, the experimental and control group participants completed a video conference check-in with a member of the research staff. During these check-ins, participants were asked to report how often they logged into CT-R to complete their exercises (experimental group) or how many workbook pages had been completed that week (control group). In addition, they were asked if they found any exercises or items too challenging or too simple. For the experimental group, as needed, the research staff modified the experimental group's homework program and documented changes. For the control group, if a participant reported that their workbook was too easy or too difficult, a correspondingly different workbook was sent to them. Details of the two interventions are provided in Table 3.

**Table 3:** Description of the intervention per TIDieR descriptions.

**Item No**.	**Item**	**TIDieR description**	
		**Experimental (constant therapy group)**	**Control (workbook group)**
1.	WHY	Self-management of home-based therapy under remote-guidance could result in an individualized therapy protocol, increased adherence, and compliance of home practice will improve language skills.	Self-management of home-based therapy under remote-guidance without the structured feedback and regimen would result in limited gains.
2.	WHAT materials	Constant Therapy-Research was used as a tailored home treatment program for each participant.	Aphasia therapy workbooks were used for home practice.
3.	WHAT procedures	For each trial in the Constant Therapy-Research software, the participant can select the answer and choose whether to use cues. Once the participant selects the response, immediate feedback is provided regarding accuracy and the participant can proceed to the next trial.	The participant can select pages of workbook to work on for therapy. No feedback is provided on accuracy of attempts on individual items in workbook.
4.	WHO PROVIDED	Speech Language Pathologists or Trained Research Assistants	Speech Language Pathologists or Trained Research Assistants
5.	HOW	Weeks 2 through 8, the experimental group participants completed a video conference check-in with a member of the research staff. During these check-ins, participants were asked to report how often they logged into CT-R to complete their exercises.	Weeks 2 through 8, the control group participants completed a video conference check-in with a member of the research staff. During these check-ins, participants were asked to report how many workbook pages had been completed that week.
6.	WHERE	Participant homes via videoconference	Participant homes via videoconference
7.	WHEN and HOW MUCH	Participants were instructed to use CT-R for at least 30 min a day and at least 5 days a week. CT-R tracked usage of the program.	Control participants were instructed to complete at least 1 exercise within the workbook at least 5 days a week.
8.	TAILORING	Each participant advanced via NPE algorithm using the library of therapy exercises (over 100,000 stimuli within 350+ levels of difficulty spanning 9 different cognitive, speech, and language domains).	Three workbooks with different levels of difficulty were offered to all participants based on participant feedback.
9.	MODIFICATIONS	_______NA______	________NA_____
10.	HOW WELL: PLANNED and ACTUAL	CT-R tracks the daily log in times and durations for therapy completion. Biweekly check-ins confirmed study adherence.	Biweekly check-ins confirmed study adherence.

### Data Reliability and Data Analysis

#### Data Entry

All assessments were scored utilizing hard copies of the WAB-R, BTACT, and SAQOL at the time of administration. Study personnel then checked and entered these scores into a shared spreadsheet and filed hardcopies into secure participant folders.

#### Data Reliability

All assessments were entered and checked for accuracy by study personnel. Two randomly selected raters from a group of four raters checked administration and scoring of the WAB-R (AQ, LQ, and CQ) on 11% of the total pre- and post-WAB assessments. Inter-rater reliability was high (Cronbach's Alpha = 0.997) with a difference score on the AQ scores to be 1.84 points, CQ scores to differ by 1.57 points, and LQ scores to differ by 1.52 points. Further, sections of the WAB-R including the Spontaneous Speech fluency and content rating scales and the Sequential Commands subtest, were discussed at length among study personnel to create standardized interpretations and scoring of participant responses. Consensus scoring across three raters was utilized for both of the Spontaneous Speech rating scales for all participants.

#### Statistical Analysis

Given unequal sample sizes, a linear mixed effects model was conducted on the primary and secondary outcomes. In all analyses, score on the specific test (WAB-AQ, LQ, etc.) was the dependent variable, group (CT-R vs. workbook) and time point (pre-treatment and post-treatment) were the fixed factors, age, and time post-stroke were entered as covariates (unless otherwise noted) and participants were entered as random factors. As follow-up analyses, RANOVAS were performed to further examine treatment-related effects in the two groups.

#### Data Availability

All individual anonymized participant data are provided in Supplementary Table 1.

#### Standard Protocol Approvals, Registrations, and Patient Consents

The study was reviewed, monitored, and approved by Pearl IRB 19-LNCO-102. All participants provided informed consent for this study following procedures described above. This project is registered in the ClinicalTrials.gov registry (NCT04488029).

## Results

### Baseline Measures

**Figure 3:**
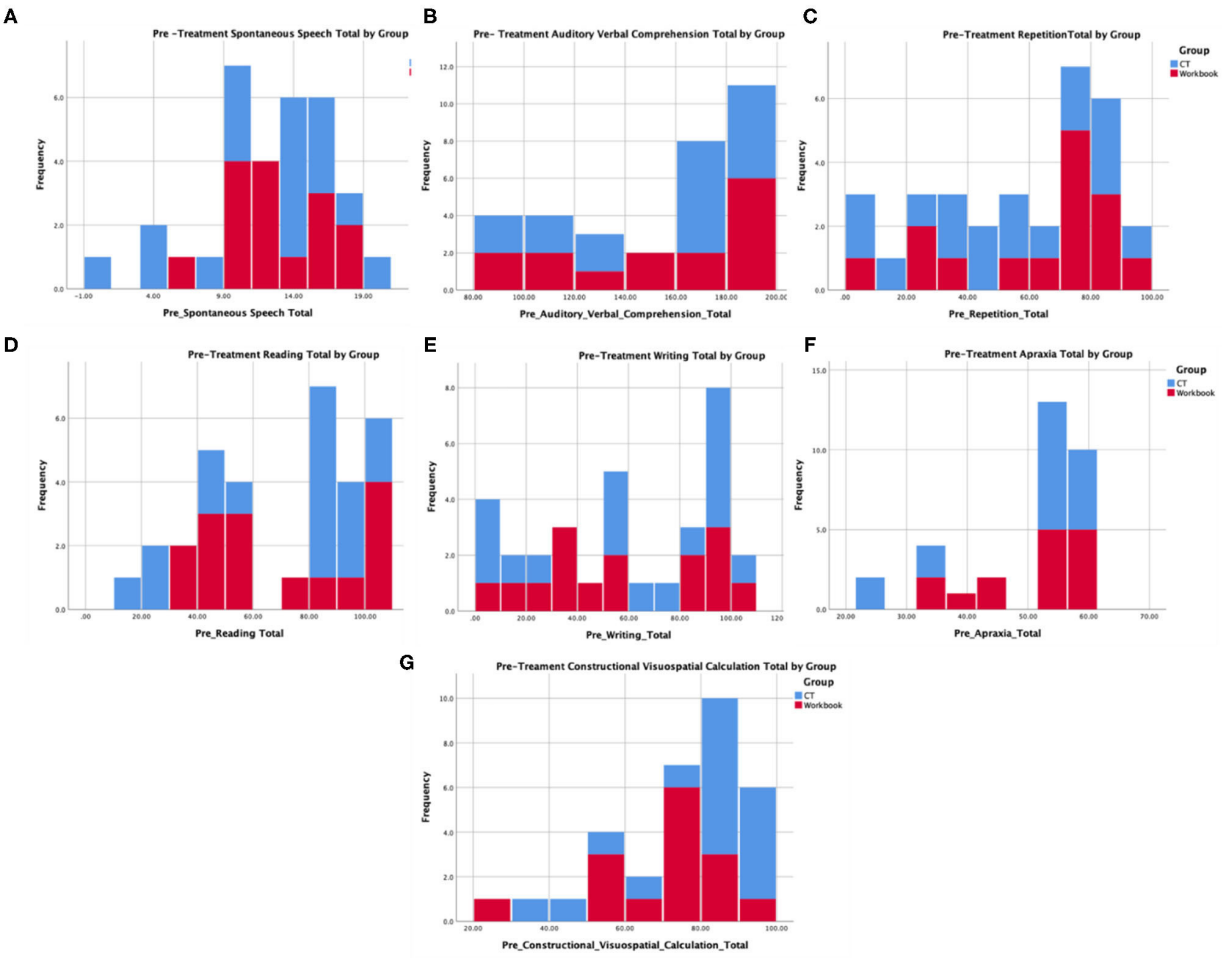
Pre-treatment language and cognitive profiles from the WAB for participants in the experimental and control group that represent **(A)** spontaneous speech, **(B)** auditory comprehension, **(C)** repetition, **(D)** reading, **(E)** writing, **(F)** apraxia, and **(G)** construction, visuospatial, and calculation.

Table 1 provides baseline demographic and assessment measures, indicating that there were no pre-existing differences between the experimental (*N* = 17) and control (*N* = 15) groups. Further, Figure 3 provides histogram profiles of specific language and cognitive domain scores from the WAB-R indicating that both groups were similar prior to the beginning of treatment. Additionally, Kruskal-Wallis *H*-tests were used due to unequal sample sizes showing no difference between the groups on specific variables (age, *p* = 0.14, time since stroke, *p* = 0.60, baseline WAB-AQ, *p* = 0.77).

### Primary Endpoint

**Figure 4:**
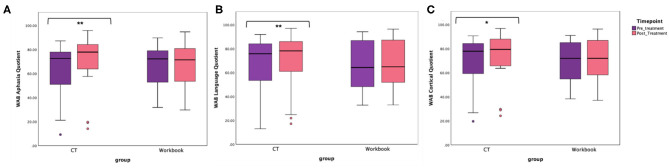
Unadjusted means for WAB-R **(A)** Aphasia Quotient, **(B)** Language Quotient, and **(C)** Cortical Quotient for the two groups (CT-R and workbook) pre-treatment and post-treatment. Significant differences are marked with *. *p < 0.05 and **p < 0.01.

The primary endpoint in the study was the average change on WAB-AQ. The CT-R group showed a higher mean point change WAB-AQ (*M* = 6.75) than the workbook group (*M* = 0.38). Using a linear mixed effects model, this change was significant at the 1% level. The significant group by time interaction indicated that on average, participants in the CT-R group had WAB-AQ scores of 6.36 points higher than the control group at follow-up than at pre-treatment that was significant (*p* < 0.01, see Tables 2, 4 and Figure 4A).

**Table 4:** Statistical analyses for primary and secondary outcomes in the study.

**Linear Mixed Model for Primary and Secondary Outcome Measures**		
	**Coefficient**	***P*****-value**
***WAB-AQ (no covariates)***		
Intercept	66.02	**<0.001**
Intervention group (vs. control group)	−4.39	0.59
Post-treatment (vs. baseline)	0.38	0.80
Intervention group* post-treatment	6.36	**<0.01**
***WAB-AQ (with covariates)***		
Intercept	105.7	**<0.001**
Intervention group (vs. control group)	−9.49	0.26
Age	−0.69	0.07
Time post-stroke	0.12	0.16
Post-treatment (vs. baseline)	0.31	0.84
Intervention group* post-treatment	6.43	**<0.01**
***WAB-LQ (with covariates)***		
Intercept	108.0	**<0.001**
Intervention group (vs. control group)	−6.83	0.44
Age	−0.71	0.08
Time post-stroke	0.11	0.23
Post-treatment (vs. baseline)	0.53	0.61
Intervention group* post-treatment	3.97	**<0.01**
***WAB-CQ (with covariates)***		
Intercept	111.5	**<0.001**
Intervention group (vs. control group)	−6.42	0.0.41
Age	−0.72	0.05
Time post-stroke	0.09	0.24
Post-treatment (vs. baseline)	0.67	0.53
Intervention group* post-treatment	4.01	**<0.05**
**Repeated Measures ANOVAs for Secondary Outcome Measures**		
	**f-ratio (error df)**	***P*****-value**
***BTACT***		
**Verbal Fluency**		
Group	2.15	0.15
Time	5.73 (27)	**0.02**
Group* Time	0.239 (27)	0.62
**Immediate Recall**		
Group	1.79 (26)	0.19
Time	1.36 (26)	0.25
Group* Time	1.36 (26)	0.25
**Digit Span Backwards**		
Group	2.48 (27)	0.12
Time	3.73 (27)	0.06
Group * Time	1.05 (27)	0.31
**Number Series**		
Group	1.55 (27)	0.22
Time	1.07 (27)	0.30
Group* Time	1.07 (27)	0.30
**Backward Counting**		
Group	0.652 (27)	0.42
Time	0.248 (27)	0.62
Group* Time	0.248 (27)	0.62
**Delayed Recall**		
Group	0.12 (27)	0.73
Time	1.42 (27)	0.24
Group * Time	0.476 (27)	0.49
***SAQOL***		
**Mean**		
Group	0.02 (27)	0.88
Time	5.92 (27)	**0.02**
Group * Time	0.886 (27)	0.35
**Physical**		
Group	0.02	0.87
Time	0.11 (27)	0.74
Group * Time	0.46 (27)	0.52
**Communication**		
Group	0.63 (27)	0.43
Time	4.33 (27)	**0.04**
Group * Time	0.04 (27)	0.83
**Psychosocial**		
Group	0.003	0.95
Time	0.15 (27)	0.70
Group * Time	1.35 (27)	0.25
**Energy**		
Group	1.00 (27)	0.32
Time	8.78 (27)	**0.006**
Group * Time	0.35 (27)	0.06

*Bold values are statistically significant*.

### Primary Endpoint With Covariates

Even though there were no significant pretreatment differences between the two groups in terms of age, time since stroke and baseline WAB-AQ, controlling for these factors in a linear mixed effects model showed that being in the CT-R group was associated with a 6.43 point increase in WAB-AQ score relative to the workbook group at follow-up than at pre-treatment. Specifically, Table 4 illustrates that the starting baseline WAB-AQ score was 105.7 (intercept), adjusted by −0.69 for every year of age, and by 0.122 for every month since stroke, participants in the CT-R group had WAB-AQ scores 6.43 higher than the workbook group at the end of treatment.

It is worth noting that the mean differences as a function of treatment for sub scores that comprise the WAB-AQ, were consistently higher for the experimental group than the control group (see Tables 2, 4), including spontaneous speech (CT-R = 1.35, workbook = −0.33), auditory comprehension (CT-R = 12.05, workbook = 4.46), repetition (CT-R = 10.12, workbook = 3.46), and naming (CT-R = 4.05, workbook, −0.46).

### Secondary Endpoints

An additional secondary endpoint was average change on the WAB-LQ. The CT-R group showed a higher mean change (*M* = 4.51) than the workbook group (*M* = 0.57) points. Table 4 shows that the effects of group, age, time from stroke, and post-treatment (vs. baseline) were not significant. The significant interaction of post-treatment relative to baseline by group, controlling for other variables, was significant, indicating that on average, participants in the CT-R group had WAB-LQ score of 3.97 points higher than the workbook group at post-treatment (Figure 4B). Again, mean differences as a function of treatment for subscores of reading were higher for the experimental group (4.00) than the control group (1.20), however, writing scores worsened for both groups (see Table 2).

**Figure 5:**
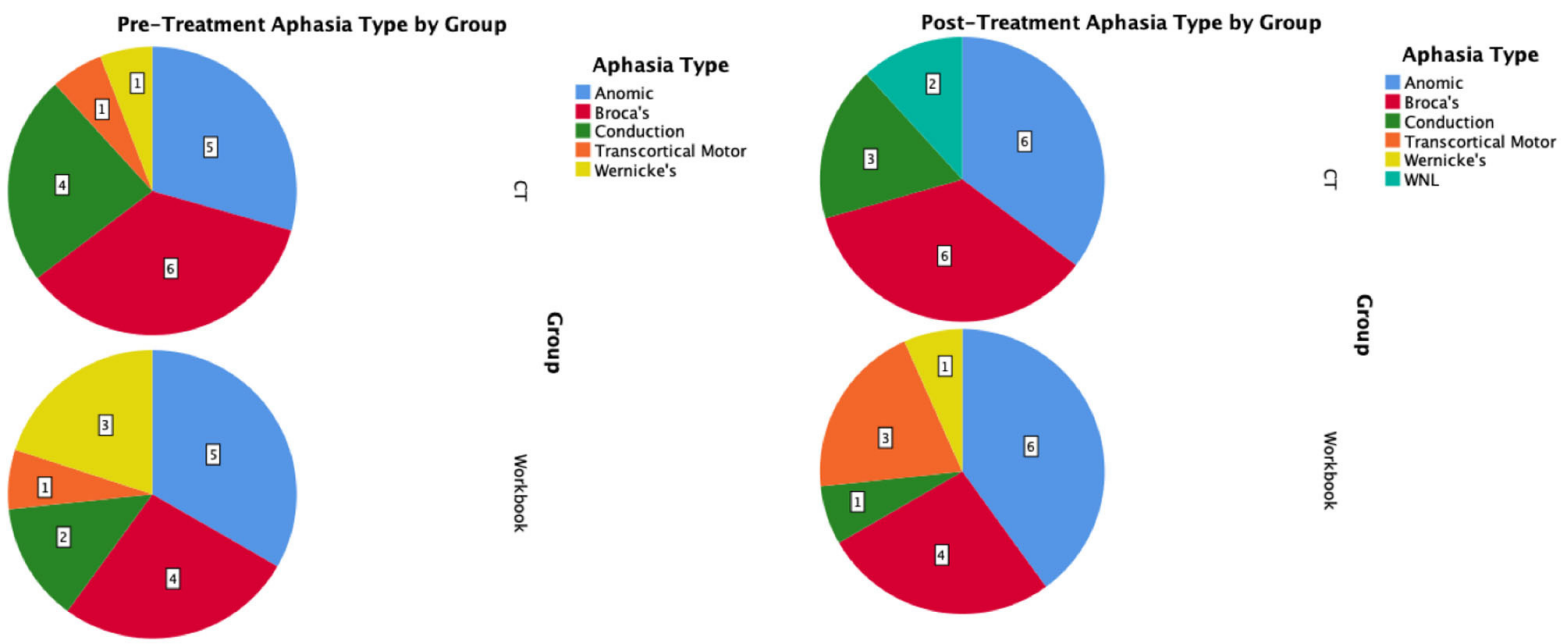
Pre-treatment and post-treatment aphasia subtypes as calculated by the WAB-R shown as pie-charts by intervention group.

The mean change on the WAB-CQ, showed that the CT-R group showed a higher mean change (*M* = 4.69) than the workbook group (*M* = 0.77). Again, only the interaction of post-treatment relative to baseline by group (controlling for other factors) was significant; participants in the CT-R group had an average WAB-CQ score of 4.01 points higher than the workbook group at the end of treatment (Figure 4C). Mean differences as a function of treatment for subscores of apraxia were higher for the experimental group (1.76) than the control group (−0.33), however, mean differences as a function of treatment for subscores of constructional and visuospatial calculation were higher for the control group (3.40) than the CT-R group (1.55) (Table 2). Finally, in addition to changes on specific subscores in the WAB, Figure 5 shows that there were qualitative changes in the aphasia subtypes (as calculated by the WAB) as a function of treatment. Specifically, in the CT-R group, while there were a range of aphasia types prior to treatment, after treatment all participants fell into one of four subtypes (Anomic, Broca's, Conduction, and Within Normal Limits). Contrastingly, the workbook group showed more subtle qualitative changes, and none of them were classified as being within normal limits. Given the small sample sizes of subcategories, no statistical analyses were computed.

**Figure 6:**
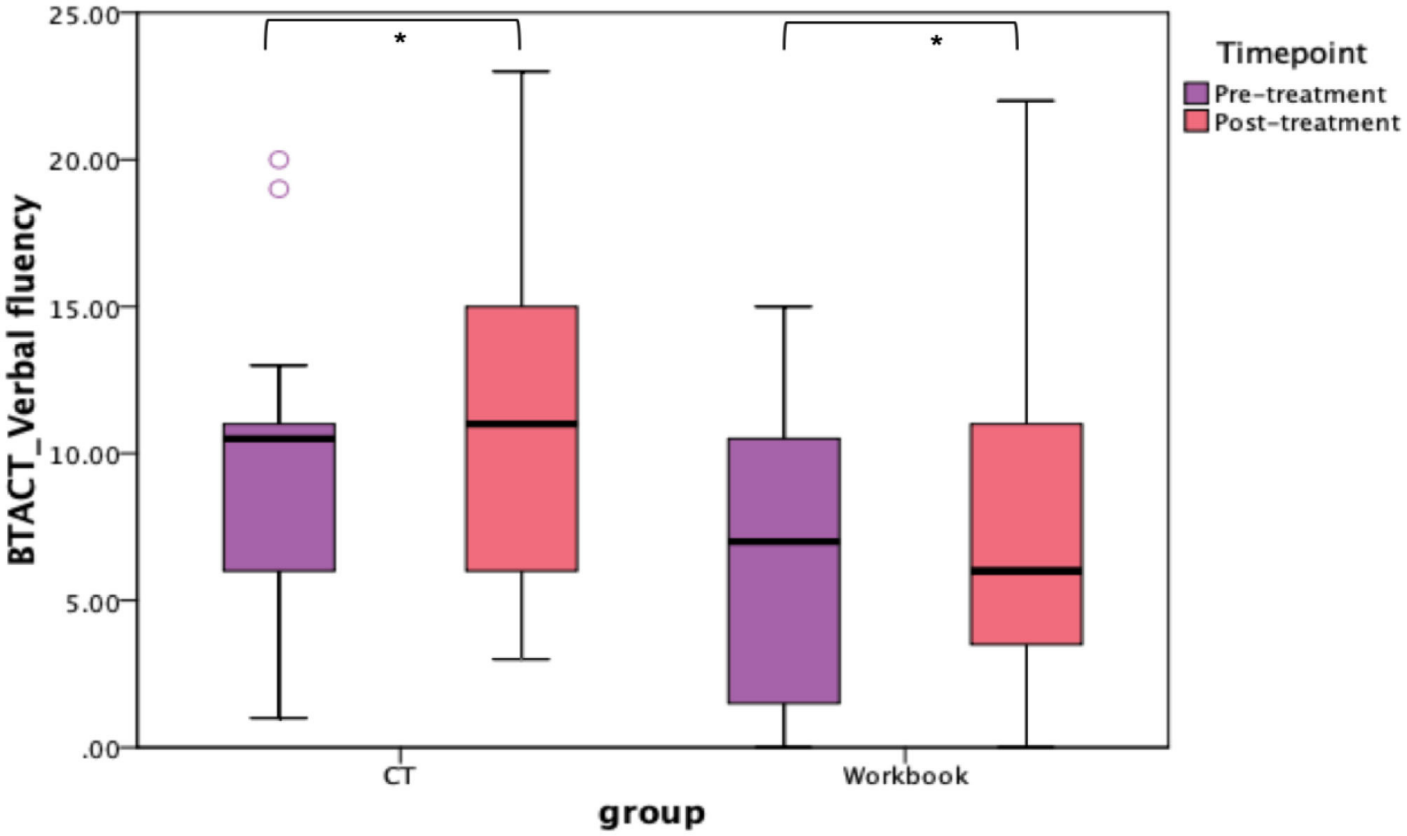
Unadjusted means for BTACT for the two groups (CT-R and workbook) pre-treatment and post-treatment. Significant differences are marked with *. *p < 0.05.

**Figure 7:**
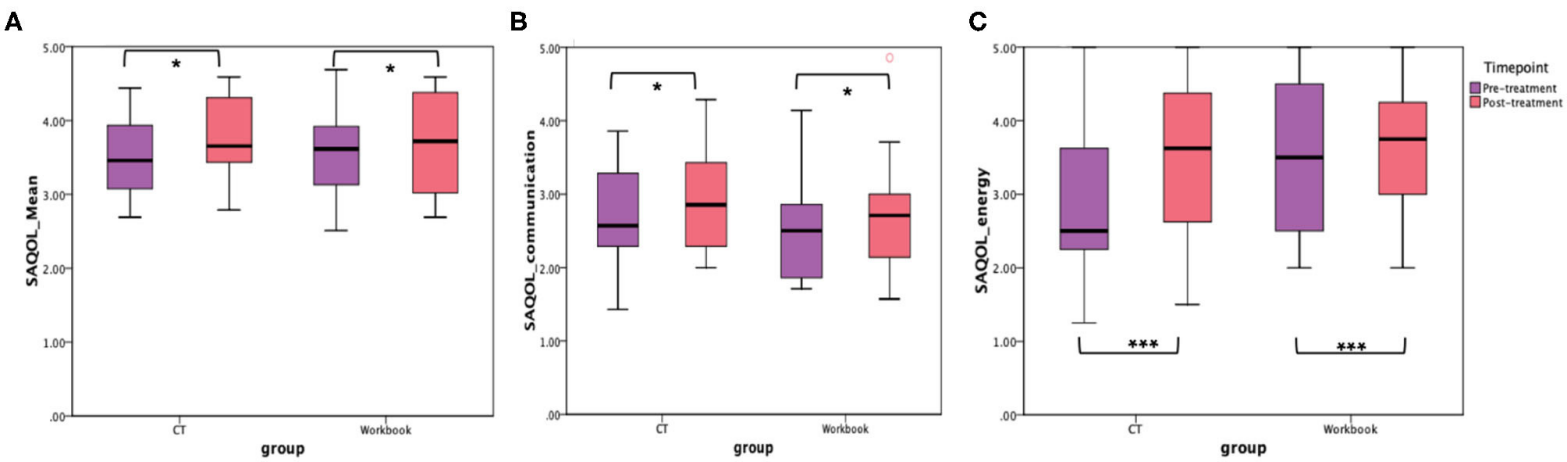
Unadjusted means for **(A)** SAQOL_mean, **(B)** SAQOL_communication, and **(C)** SAQOL_energy for the two groups (CT-R and workbook) pre-treatment and post-treatment. Significant differences are marked with *. *p < 0.05 and ***p = 0.006.

In addition to the WAB-R, BTACT, and the SAQOL-39 were also examined (see Figures 6, 7). The mixed effects models for these two measures were not significant for either the main effects or the interaction effects. Therefore, follow-up repeated measures ANOVAs with scores on each of the subtests as the dependent variable, and time (pre-treatment, post-treatment), group (CT-R vs. workbook group), and the interaction between time and group were conducted. Table 4 reflects all the analyses, the F-ratios and the *p*-values. On the BTACT, only the subtest of verbal fluency showed a significant effect of time, but no significant effects of group or interaction between group and time. On the SAQOL, the overall mean showed a significant improvement as a function of time, as the main effect of group or the interaction between group and time was not significant. Similar results were observed for SAQOL_communication and SAQOL_energy sub-scores, indicating that both groups showed improvements as a function of treatment. The remaining contrasts were not significant.

Finally, to examine the potential influence of demographic variables on the primary outcome measure, bivariate correlations revealed a significant moderate negative relation between age and difference on the post-pre WAB-AQ score (*r* = −0.45, *p* < 0.01), but no significant relation between time since stroke in months and difference on the post-pre WAB-AQ score (*r* = −0.07, *p* > 0.05), and between education in years and difference on the post-pre WAB-AQ score (*r* = −0.09, *p* > 0.05).

## Discussion

Currently, standard of care (SOC) for speech therapy involves a stepped approach to rehabilitation in the days, weeks, months, and years following stroke. In general, at each phase following a stroke, there are different SOCs (45). These phases can be described as “acute” (typically the first 24–48 h after a stroke, where the priority is saving a life), “in-patient” (when the patient is recovering, often with medical monitoring, and intense multidisciplinary care), “out-patient” (when living at home but receiving periodic care from healthcare professionals), and “post-discharge” (when no longer under the care of clinical teams). It is in the post-discharge phase that SOC dictates that patients undergo self-directed maintenance. Self-directed maintenance may include the application of learned strategies to daily functional communication exchanges and/or identification of activities or exercises that will allow for practice of the skill area. As noted in the introduction, the state of today's SOC results in the overwhelming majority of patients not receiving the benefit of consistent one-on-one therapy after the first month following their stroke due to structural barriers that preclude extension of traditional one-on-one therapy at a frequency and duration likely associated with optimal outcomes.

The present study was the first virtual language/cognitive rehabilitation trial for individuals with post-stroke aphasia. Further, this study joins other recent trials (20) that provide evidence for digitally-based language therapy for post-stroke patients. This Phase II trial showed that individuals who practiced CT-R at home with biweekly check-ins showed an average of 6.43 points greater change on WAB-AQ scores at the end of treatment relative to a control group that practiced workbooks at home and also received biweekly check-ins, even after controlling for age and time post-stroke for participants in the two groups. Importantly, the CT-R group showed a mean improvement of 6.75 points on the WAB-AQ, a change that is above the 5 point threshold for clinically meaningful improvement in speech-language ability (46–48), compared to 0.38 for conventional workbook intervention. Notably, the CT-R group outperformed the control group at the end of the treatment program on WAB-LQ (4.51 points for the CT-R group) and the WAB-CQ (4.69 points for the CT-R group). Changes on the subscores of the WAB subtests were consistently higher for the CT-R group than the workbook group, including on spontaneous speech, auditory comprehension, repetition, naming, reading and apraxia. Interestingly, writing scores worsened slightly for both groups and construction, visuospatial and calculation increased for the workbook group more than the experimental group. Decreases in the writing subtests for both groups may be reflective of the reality that CT-R writing practice is done on a tablet and is different from handwriting; and the workbook group may have not practiced writing consistently. It is not completely clear why the workbook group improved more on the constructional, visuospatial, and calculation subtests, but further inspection of participant data suggests that the workbook group improved more on the calculation sections of the WAB. Interestingly, when examining any changes in aphasia subtypes as a function of the treatment, results showed that CT-R made more discernable shifts in their aphasia subtypes, subsequent to improved WAB subscores than the workbook group. Notably, post-treatment, two participants were classified as being within normal limits as per the WAB in the CT-R group, a similar shift was not observed in the workbook group.

Participants in the CT-R group logged into the software program at least 5 days per week, practiced a prescribed number of therapy exercises and received instant feedback on the accuracy for each item. In contrast, the workbook group received physical workbooks to practice, were instructed to practice multiple pages, and importantly, instant feedback was not provided. Therefore, it is possible that the impairment-based drill therapy with feedback targeted in the CT-R software facilitated transfer of similar performance on the domains of language and cognitive function tested by WAB.

Another observation is the difference in the treatment approaches between the experimental and control groups. CT-R was designed to progress the participant through targeted therapy tasks based on their performance. For example, if a participant passed an exercise easily, they would automatically be given a harder task targeting the same skill or domain in the next session. Alternatively, if they appeared to struggle with an exercise, then upon the next login, CT-R would present an easier task targeting the same skill. This automatic calibration of task delivery was designed as part of the software's algorithm with an optional oversight from a study staff. The experimental group, using the CT-R program, also had the added benefit of a study staff being able to manually modify or update their homework program based on participant feedback. The control group, while using the workbooks could provide feedback regarding the exercises, but could not have the study staff modify, update, or change the homework tasks remotely. These inherent differences in how the treatment program was tailored for each individual participant in the experimental group relative to the control group may have also contributed to differences in the primary outcomes for the two groups.

Compared to the WAB, the critical interaction group by time was not significant for the SAQOL-39 or BTACT in the linear mixed effect models. Instead, repeated measures ANOVAs that compared the two groups as a function of time showed that both the BTACT_verbal fluency and specific SAQOL measures (i.e., SAQOL_mean, SAQOL_communication and SAQOL_energy) improved for both groups as a function of treatment, indicating that participation in the 10 week remote intervention, independent of the type of treatment, resulted in gains on verbal fluency on the BTACT and quality of life perception on the SAQOL. Apart from the main difference in the mode of therapy exercises practiced, the bi-weekly check-ins with the study staff and the level of flexibility in therapy session practice were identical between the two groups. Therefore, it is possible that the frequent interaction with the study staff who provided feedback about therapy progress and the consequent accountability may have had the same faciliatory effects for both groups. Relatedly, compliance with attendance at bi-weekly check-ins was high across both groups, reinforcing findings that telerehabilitation access decreased missed appointments rates (49). By decreasing barriers due to transportation, commute time, and time out of work, teletherapy provides patients with a more flexible option that ultimately improves engagement with the therapy process. It is important to note that these check-ins were completed completely virtually over videoconference; both as we handle the challenges of COVID-19 and as we look to the future of telepractice, this is encouraging data suggesting that virtual interaction continues to be motivating and engaging for patients.

Nonetheless, the lack of a greater improvement on the secondary outcome measures in the CT-R group vs. the workbook group requires further discussion. It should be noted that the mean difference in the SAQOL-39 ratings for the submeasures ranged from 0.24 to 0.60 (SAQOL_Mean, SAQOL_energy, respectively) for the CT-R group relative to −0.05 to −0.36 (SAQOL_communication, SAQOL_psychosocial, respectively) for the workbook group. These differences for the CT-R group are comparable to 0.33 difference in a study examining the effect of phonomotor treatment on word retrieval (50), hence, contextualizing the gains on this measure in the CT-R group. The BTACT was selected due to its remote administrability, however, there are no studies that report BTACT as an outcome measure for treatment, thus limiting any points of comparison. Additionally, the BTACT requires auditory comprehension and verbal expression, thereby limiting its sensitivity to determine isolated improvements in cognitive function. This hypothesis is further supported by the evidence that participants in the CT-R group increased in the WAB-CQ (a more non-linguistic measure of cognitive function) by 4.97 points higher than the control at follow-up, indicating that improvement in cognitive function was observed by a more non-linguistic measure.

Another interesting but secondary finding of this trial is evidence that PWA can make gains in their language and cognitive skills even in the chronic phase of rehabilitation. While most recovery is expected to occur in the first few months after the stroke (5, 51), this study demonstrates that it is possible to improve language skills in this population even multiple years post-stroke. The average time post-stroke for the participants in the experimental group of this study was 46 months. Yet, there was no significant correlation between time post-stroke and the degree of gains made by patients, indicating that recovery can continue for many years post-stroke. There was a moderate negative correlation between age and improvement on the WAB-R for AQ scores, which does indicate that older patients tended to make fewer gains. Conversely, while some participants were well into their 80's, they were still able to access and manipulate the provided technology, given instruction and support from study personnel, dispelling a common myth that older adults are less able to utilize technology.

While the results from this study are encouraging regarding the implementation of virtual trials, teletherapy as a service delivery model and the use of digital therapeutics like CT-R, there were some limitations to the study. Thirty-two participants were a modest sample size for a study of this patient population, and it is unclear whether these results generalize beyond this study to other similar studies, as well as to other implementations of teletherapy and digital therapeutics. Additionally, there were some practical constraints and barriers to conducting the study. First, as the target population ranged from mild-severe/profound language impairment, it was both critical and necessary for all participants to have a caregiver present during the initial onboarding into the study and pre/post assessments. Nonetheless, even participants with a severe language impairment were able to initiate and complete their homework programs once education and training was provided. Additionally, logistical considerations such as shipping and tracking of materials, and troubleshooting technology, required ongoing time and attention from the study team throughout the trial. Recruitment practices also had to be adjusted to better fit a virtual trial, and instead of the traditional recruitment through a clinical setting, social media and targeted advertising to educate potential participants were implemented recruit them into the study.

While more studies are needed, these results provide encouraging data supporting the efficacy of digitally-based therapeutics, teletherapy, and virtual trial administration. Given that this is the first completely virtual, digital therapeutic treatment study with both assessments and therapy provided remotely, several conclusions can be drawn. First, completely virtual randomized control trials can be performed with checks and balances in place such as weekly check-ins with patients. Second, all the chosen assessments were verified in previous studies for administration in remote assessments and were implementable in a clinical trial. Third, the feasibility of such a trial indicates a novel approach to conduct telerehabilitation studies in an asynchronized format (i.e., participants practice their therapy when it is convenient for them, and without the presence of a clinician) with successful outcomes. Finally, this trial provides evidence that remote assessment and intervention of post-stroke aphasia is both effective and aligned with the ever-shifting needs of how people access care. Participants in this study were located across the United States and Canada and completed the study without issue, suggesting that telehealth services such as these can reduce the geographic challenges that many patients with aphasia face when seeking therapy.

## Data Availability Statement

The raw data supporting the conclusions of this article will be made available by the authors, without undue reservation.

## Ethics Statement

The studies involving human participants were reviewed and approved by Pearl IRB (19-LNCO-102). The patients/participants provided their written informed consent to participate in this study.

## Author Contributions

MB, ED, SS, VA, and SK designed and conceptualized study. MB, JP, and SK drafted manuscript for intellectual content. SS drafted and documented IRB protocol. MB, JP, ED, SS, LT, SL, VA, and SK revised manuscript. VA was oversight of technology implementation. MB, JP, and SS were major role in data acquisition. SL was oversight of study conduct, data management, and reporting. JP, SS, LT, SL, and SK analyzed and interpreted the data. All authors contributed to the article and approved the submitted version.

## Conflict of Interest

Authors were employed by The Learning Corp, makers of Constant Therapy, during study administration.

## Footnotes

^1^CT-R was referred to as “PCT” within IRB documentation and ClinicalTrials.gov registration. It was internally renamed as CT-R for clarity and is otherwise identical to PCT.
